# Association of Preoperative Platelet-Activating Factor and Postoperative C-Reactive Protein with Inflammatory Burden and Early Outcomes After Major Cardiac Surgery

**DOI:** 10.3390/biomedicines14051149

**Published:** 2026-05-19

**Authors:** Adrian Stef, Gabriel Cismaru, Aurelia Georgeta Solomonean, Nadina Tintiuc, Tudor-Mihai Magdaș, Alexandru Oprea

**Affiliations:** 1 Anesthesia and Intensive Care 2 Discipline, “Iuliu Hațieganu” University of Medicine and Pharmacy, Victor Babes Nr 8 Street, 400012 Cluj-Napoca, Romania; stef.adrian@yahoo.com (A.S.); aureliageorgeta@gmail.com (A.G.S.); ntintiuc@yahoo.com (N.T.); tmmagdas@gmail.com (T.-M.M.); 2 Clinical Department of Anesthesia and Intensive Care, Heart Institute “Niculae Stăncioiu”, “Iuliu Hațieganu” University of Medicine and Pharmacy, Motilor 19-21, 400001 Cluj-Napoca, Romania; 3 4th Department of Internal Medicine, Cardiology Rehabilitation, “Iuliu Hațieganu” University of Medicine and Pharmacy, Victor Babes Nr 8 Street, 400012 Cluj-Napoca, Romania; 4 Romania National Institute for Research and Development of Isotopic and Molecular Technologies, 67-103 Donat Street, 400293 Cluj-Napoca, Romania; 5 Department of Surgery, “Iuliu Hațieganu” University of Medicine and Pharmacy, 400000 Cluj-Napoca, Romania; alexandru_oprea2002@yahoo.com; 6 Cardiovascular Surgery Department, Heart Institute “Niculae Stăncioiu”, “Iuliu Hațieganu” University of Medicine and Pharmacy, Motilor 19-21, 400001 Cluj-Napoca, Romania

**Keywords:** major cardiac surgery, platelet-activating factor, C-reactive protein, cardiopulmonary bypass, vasoplegia syndrome

## Abstract

**Background:** Major cardiac surgery with cardiopulmonary bypass (CPB) induces a systemic inflammatory response that contributes to postoperative organ dysfunction and hemodynamic instability. While C-reactive protein (CRP) is a well-established downstream marker of postoperative inflammation, the upstream determinants of interindividual variability in inflammatory burden are not fully understood. Platelet-activating factor (PAF) is a potent inflammatory mediator implicated in platelet activation, endothelial dysfunction, and vascular dysregulation, but its role in modulating postoperative inflammation and clinical outcomes after cardiac surgery has not been fully characterized. **Methods:** We conducted a retrospective observational study of 87 patients undergoing major cardiac surgery with CPB. Preoperative plasma PAF levels and postoperative CRP concentrations were measured, and patients were stratified according to postoperative CRP severity. Associations between PAF, inflammatory response, postoperative vasoactive–inotropic requirements, recovery parameters, acute kidney injury, and mortality were assessed using correlation analyses, multivariable regression models, and receiver operating characteristic curve analyses. **Results:** Preoperative PAF levels increased progressively across postoperative CRP strata (*p* < 0.001) and were strongly associated with postoperative CRP concentrations in both univariate and multivariable analyses. Specifically, each 1000 pg/mL increase in preoperative PAF was associated with an adjusted increase of 36.0 mg/L in postoperative CRP (β = 36.0; *p* < 0.001). Each 1000 pg/mL increase in preoperative PAF was associated with an adjusted increase of approximately 36 mg/L in postoperative CRP. Elevated PAF was also associated with increased intermediate postoperative vasoactive–inotropic requirements and a modest increase in hospital length of stay (r = 0.25, *p* = 0.023). However, neither PAF nor CRP independently predicted AKI or mortality after adjustment for clinical variables. Discriminative performance for mortality was modest for both biomarkers. **Conclusions:** Preoperative platelet-activating factor was strongly associated with postoperative inflammatory burden and early hemodynamic instability following major cardiac surgery. Although PAF and CRP were not independent predictors of adverse outcomes, they may help identify a biologically vulnerable phenotype characterized by exaggerated inflammatory and vascular responses to surgical stress. These findings support further investigation of platelet-mediated inflammatory pathways as targets for perioperative risk stratification and mechanistic research.

## 1. Introduction

Major cardiac surgery with cardiopulmonary bypass (CPB) is accompanied by a profound systemic inflammatory response that contributes to postoperative hemodynamic instability, organ dysfunction, and delayed recovery [1,2]. This response is driven by multiple interacting mechanisms, including blood–surface contact within the extracorporeal circuit, ischemia–reperfusion injury, endothelial activation, and complex platelet–leukocyte interactions [1,3]. Despite advances in surgical technique and perioperative management, inflammation-related complications remain a major determinant of postoperative morbidity.

C-reactive protein (CRP) is widely used as a clinical marker of postoperative inflammation and has been consistently associated with adverse outcomes after cardiac surgery, including prolonged recovery and increased complication rates [4,5,6,7]. However, CRP reflects downstream activation of inflammatory pathways and does not fully capture the upstream biological processes that determine the magnitude of the postoperative inflammatory response. Notably, substantial interindividual variability in CRP elevation is observed even among patients undergoing similar procedures, suggesting the presence of pre-existing biological susceptibility rather than differences in surgical complexity alone [6,8].

Platelet-activating factor (PAF) is a potent phospholipid mediator with well-established roles in platelet activation, leukocyte recruitment, endothelial dysfunction, and amplification of inflammatory signaling [9,10,11]. PAF has been implicated in cardiovascular inflammation, microvascular dysfunction, and nitric oxide dysregulation—processes that are central to the pathophysiology of CPB and postoperative vasoplegia [10,12,13]. Experimental and translational studies have demonstrated that PAF can amplify inflammatory cascades and contribute to vascular hyporesponsiveness, but its clinical relevance in the perioperative cardiac surgery setting remains incompletely defined [9,10].

Although prior studies have examined inflammatory biomarkers in cardiac surgery populations, most have focused on postoperative measurements or downstream markers such as CRP, interleukins, or complement activation products [4,7,14]. In contrast, the potential role of preoperative platelet-mediated inflammatory activation as a determinant of postoperative inflammatory burden and clinical susceptibility has received comparatively little attention. Identifying upstream mediators that predispose patients to exaggerated inflammatory responses may provide mechanistic insight beyond conventional risk factors and operative variables [3,8].

Given the profound complexity of the systemic inflammatory response following CPB [15,16,17,18], this study was designed as an exploratory investigation to translate established pathophysiological reasoning regarding the role of platelet-activating factor (PAF) in the inflammatory cascade into the clinical context of major cardiac surgery. We hypothesized that elevated preoperative PAF identifies a biological phenotype predisposed to an exaggerated postoperative inflammatory response and subsequent hemodynamic instability, independent of operative complexity or baseline cardiac function.

Our primary aim is to explore whether identifying these upstream mediators, such as PAF, can help clinicians anticipate the physiological needs following major cardiac surgery. Consequently, the objectives of this study were to: (1) Examine the association between preoperative PAF and postoperative CRP as a measure of inflammatory burden; (2) Evaluate the relationship between PAF and postoperative hemodynamic support; (3) Assess the prognostic utility of PAF and CRP for adverse postoperative outcomes, including AKI and mortality, in patients undergoing major cardiac surgery with CPB.

## 2. Materials and Methods

### 2.1. Study Design and Population

This retrospective observational study included adult patients who underwent major cardiac surgery with CPB at the Heart Institute “Niculae Stăncioiu,” Cluj-Napoca, Romania, between January 2021 and December 2022. This study was approved by the Ethics Committee of the “Iuliu Hațieganu” University of Medicine and Pharmacy (approval no. 259, 28 September 2023) and conducted in accordance with the Declaration of Helsinki. Due to the retrospective nature of this study and use of de-identified clinical data, informed consent was waived. Inclusion criteria were age ≥ 18 years, performance of open cardiac surgery requiring CPB, and availability of complete perioperative and laboratory data in the institutional registry. Exclusion criteria included emergency, minimally invasive and off-pump surgeries, transcatheter procedures, and incomplete clinical or laboratory records.

### 2.2. Data Collection

Demographic and clinical data were extracted from electronic medical records and included age, sex, body weight, height, body mass index (BMI), cardiac diagnosis, type of surgical procedure, preoperative left ventricular ejection fraction, and comorbidities including diabetes mellitus. Perioperative variables included CPB duration, aortic cross-clamp time, duration of mechanical ventilation, length of intensive care unit (ICU) stay, and total hospital length of stay. Postoperative complications recorded included hemodynamic instability requiring vasoactive or inotropic support, arrhythmias, AKI, infectious complications, neurological events (ischemic or hemorrhagic stroke), and in-hospital mortality.

### 2.3. Biomarker Measurements

Preoperative blood samples for platelet-activating factor (PAF) measurement were collected prior to surgery. Plasma was separated by centrifugation and stored according to manufacturer recommendations until analysis. PAF concentrations were measured using a commercially available competitive enzyme-linked immunosorbent assay (ELISA) kit (St John’s Laboratory Ltd., London, UK; Catalog #STJE0011249), with colorimetric detection; optical density was inversely proportional to PAF concentration. The assay demonstrated a sensitivity of 224 pg/mL and a detection range of 468–30,000 pg/mL, with intra- and inter- assay coefficients of variation (CV) < 10%. Results were expressed in pg/mL. Postoperative C-reactive protein (CRP) concentrations were obtained from routine laboratory measurements, with the reported value representing the peak concentration during the early postoperative period (48–72 h). For stratified analyses, patients were classified according to postoperative peak CRP values into four categories: normal (0–10 mg/L), mildly increased (10–40 mg/L), moderately increased (40–200 mg/L), and severely increased (>200 mg/L).

### 2.4. Outcome Definitions

Acute kidney injury (AKI) was defined according to KDIGO criteria and postoperative clinical diagnosis was documented in the medical records. Mortality was defined as all-cause in-hospital death. A composite adverse outcome was defined as the occurrence of any of the following: in-hospital mortality, AKI, or stroke. Postoperative hemodynamic support was quantified using the maximum vasoactive–inotropic score (VIS), which was calculated using the highest doses of vasoactive and inotropic medications administered during the first 24 h after ICU admission.

VIS = dopamine (μg/kg/min) + dobutamine (μg/kg/min) + 100 × epinephrine (μg/kg/min) + 10 × milrinone (μg/kg/min) + 10,000 × vasopressin (U/kg/min) + 100 × norepinephrine (μg/kg/min) + 50 × levosimendan (μg/kg/min)

### 2.5. Statistical Analysis

Statistical analysis was performed using SPSS software version 23.0 (IBM Corp., Chicago, IL, USA). Given the exploratory and hypothesis-generating nature of this study, a formal a priori power calculation was not performed. Continuous variables are presented as mean ± standard deviation or median with interquartile range, as appropriate, while categorical variables are expressed as frequencies and percentages. Comparisons between two groups were performed using the independent-samples t-test or Mann–Whitney U test, depending on data distribution. Comparisons across multiple CRP strata were conducted using one-way analysis of variance (ANOVA). Categorical variables were compared using the chi-square test. Associations between continuous variables were evaluated using correlation analysis and scatter plots. Multivariable linear regression was used to assess predictors of postoperative CRP levels. Logistic regression analyses were performed to identify independent predictors of adverse outcomes. Results are reported as odds ratios (ORs) with 95% confidence intervals (CIs). Receiver operating characteristic (ROC) curve analysis was used to evaluate the discriminatory performance of PAF and CRP for prediction of postoperative mortality. All statistical tests were two-tailed, and a *p*-value < 0.05 was considered statistically significant. Given the exploratory and hypothesis-generating nature of these secondary analyses, formal adjustments for multiple comparisons were not performed; therefore, results should be interpreted with consideration of the potential risk for Type I error.

## 3. Results

A total of 87 patients undergoing major cardiac surgery were included (61 men and 26 women; mean age: 64.9 years). Surgical procedures included coronary artery bypass grafting (n = 30), valve surgery (n = 44), aortic surgery (8), and combined procedures (n = 5).

### 3.1. CRP-Stratified Analysis

Patients were stratified into four groups according to postoperative C-reactive protein (CRP) levels: normal (0–10 mg/L), mildly increased (10–40 mg/L), moderately increased (40–200 mg/L), and severely increased (>200 mg/L). Baseline demographic characteristics and perioperative variables—including age, sex, body mass index, CPB duration, aortic cross-clamp time, mechanical ventilation duration, ICU stay, and hospital length of stay—did not differ significantly among CRP groups (all *p* > 0.20) (Table 1). Preoperative left ventricular ejection fraction was also comparable across groups (*p* = 0.23).

In contrast, preoperative platelet-activating factor (PAF) levels differed significantly across CRP strata, demonstrating a progressive increase with increasing postoperative CRP severity (*p* < 0.001), with the highest PAF concentrations observed in patients with severe CRP elevation (Table 1).

### 3.2. Association Between PAF and Postoperative Inflammation

**Figure 1:**
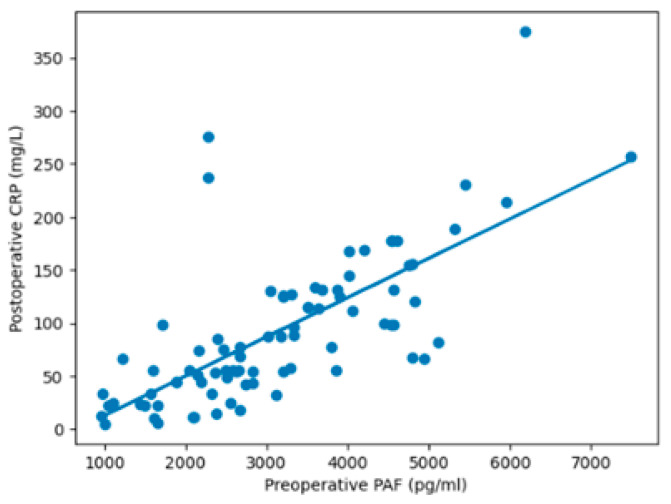
Linear association between preoperative platelet-activating factor (PAF) and postoperative C-reactive protein (CRP) based on paired individual data. Scatter plot illustrating the relationship between preoperative PAF levels and postoperative CRP concentrations, with fitted linear regression line. Increasing PAF levels are associated with progressively higher postoperative CRP values, indicating a strong positive linear relationship.

To evaluate the relationship between biomarkers at the individual level, a paired analysis was conducted using each patient’s preoperative PAF and their corresponding peak postoperative CRP concentrations. Preoperative PAF levels were strongly and positively associated with postoperative CRP concentrations (Figure 1). In multivariable linear regression analysis utilizing individual-patient data, with CRP as a continuous outcome, PAF remained independently associated with postoperative inflammation after adjustment for age, body mass index, and CPB duration. Specifically, for every 1000 pg/mL increase in PAF, there was an adjusted increase of 36.0 mg/L in CRP (β = 36.0; 95% CI 28.0–45.0; *p* < 0.001).

### 3.3. Hemodynamic Support and Recovery Parameters

Higher preoperative PAF levels were associated with increased postoperative vasoactive–inotropic requirements, with a significant positive correlation between PAF and intermediate postoperative VIS (Spearman ρ = 0.25, *p* = 0.023).

PAF showed a weak, non-significant correlation with mechanical ventilation duration (Spearman r = 0.14, *p* = 0.21). Patients with elevated PAF (>3033 pg/mL) required longer ventilation compared with those with lower PAF levels (7.3 vs. 5.3 h), with a trend toward significance (*p* = 0.060). Elevated PAF was also associated with a modest but statistically significant increase in total hospital length of stay (11.0 vs. 10.4 days, *p* = 0.037), while ICU length of stay did not differ significantly between PAF groups.

**Figure 2:**
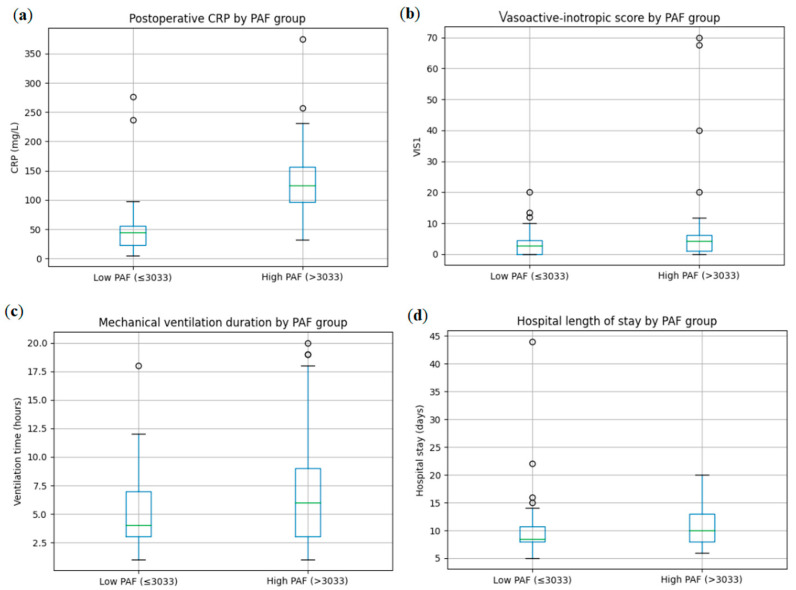
Stratification of postoperative inflammatory and clinical outcomes according to preoperative platelet-activating factor (PAF) levels. Box plots illustrate postoperative C-reactive protein (CRP), intermediate vasoactive-inotropic score (VIS), mechanical ventilation duration, and hospital length of stay in patients with low (≤3033 pg/mL) and high (>3033 pg/mL) preoperative PAF levels. (**a**) shows clear rightward and upward shift in CRP distribution in the high PAF (>3033 pg/mL) group; (**b**) higher median VIS in the high-PAF group; (**c**) higher median ventilation time in high-PAF patients; (**d**) Slight upward shift in hospital stay for high-PAF patients.

Stratification of outcomes by PAF levels demonstrated consistent distributional shifts across postoperative CRP, VIS, ventilation duration, and hospital stay, indicating that higher preoperative platelet activation identifies patients with greater postoperative inflammatory burden, increased hemodynamic support requirements, and delayed recovery (Figure 2).

### 3.4. AKI and Mortality Analyses

During the study period, 19 cases of AKI (16.53%) and 6 in-hospital deaths (incidence 4.35%) were recorded. In multivariable logistic regression analysis for AKI, age and body mass index were independently associated with AKI risk, whereas neither PAF nor CRP remained significant predictors after adjustment. For the AKI multivariable model, including five predictor variables (PAF, CRP, age, BMI, and CPB duration), the ratio of events per variable was 3.8 (Table 2).

**Figure 3:**
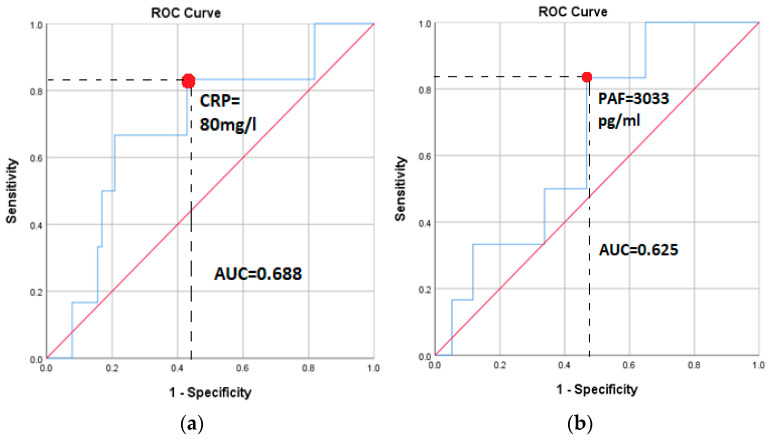
Receiver–operating characteristic (ROC) curve analysis of preoperative platelet-activating factor (PAF) and postoperative C-reactive protein (CRP) for prediction of postoperative mortality after major cardiac surgery. (**a**) PAF: AUC = 0.625; a cutoff value > 3033 pg/mL yields 83% sensitivity and 53% specificity. (**b**) CRP: AUC = 0.688; a cutoff value > 80 mg/L yields 83% sensitivity and 56% specificity.

ROC curve analysis demonstrated modest discriminatory ability of both biomarkers for postoperative mortality. The area under the curve was 0.625 for PAF and 0.688 for CRP. A PAF cutoff > 3033 pg/mL and a CRP cutoff > 80 mg/L each yielded high sensitivity (83%) but limited specificity, indicating limited stand-alone prognostic utility (Figure 3).

### 3.5. Postoperative Outcomes and Complications

Postoperative outcomes and complications across the C-reactive protein (CRP) strata are summarized in Table 3. While individual event rates for major complications such as mortality, stroke, and acute kidney injury (AKI) were relatively low in this cohort, the distribution of these outcomes demonstrated no significant differences between the CRP groups (all *p* > 0.05). The incidence of hemodynamic instability requiring support was assessed via the VIS as detailed in previous sections.

## 4. Discussion

### 4.1. Principal Findings

In this retrospective observational study of patients undergoing major cardiac surgery with CPB, we demonstrate a strong and consistent association between preoperative platelet-activating factor (PAF) levels and the magnitude of the postoperative inflammatory response, as reflected by C-reactive protein (CRP). Our results indicate that preoperative PAF is independently associated with CRP concentrations even after adjustment for relevant clinical variables and that PAF was also associated with increased postoperative vasoactive–inotropic requirements (VIS) and a modest increase in total hospital length of stay. These findings suggest that preoperative platelet-mediated inflammatory activation may identify a biological vulnerability characterized by exaggerated inflammatory and vascular responses to surgical stress, rather than functioning as a stand-alone prognostic marker for adverse postoperative outcomes.

In contrast, neither PAF nor CRP independently predicted AKI or mortality after multivariable adjustment, and their prognostic discrimination for mortality was modest.

### 4.2. Preoperative PAF and Postoperative Inflammatory Burden

Cardiac surgery with CPB induces a complex systemic inflammatory response driven by blood–surface contact within the extracorporeal circuit, ischemia–reperfusion injury, endothelial activation, and platelet–leukocyte interactions [1,2,3,19,20,21,22]. CRP is a well-established downstream marker of this response and has been consistently associated with postoperative morbidity and delayed recovery [4,5,6,7]. However, substantial interindividual variability in postoperative CRP elevation has been reported even among patients undergoing similar procedures, suggesting that pre-existing biological susceptibility plays an important role beyond surgical complexity alone [6,7,8,23,24]. While a significant portion of our cohort underwent procedures involving cardiotomies, the predictive value of preoperative PAF appears robust across these different surgical techniques, likely due to this dominant and shared inflammatory driver of CPB.

In this context, PAF represents a biologically plausible upstream mediator. PAF is a potent phospholipid involved in platelet activation, leukocyte recruitment, endothelial dysfunction, and amplification of inflammatory signaling [9,10,11,25,26,27]. Experimental and translational studies have demonstrated that PAF can amplify inflammatory cascades and promote nitric oxide dysregulation, microvascular dysfunction, and vascular hyporesponsiveness [10,12,13,28,29]. The strong, dose-dependent association observed in the present study between preoperative PAF and postoperative CRP supports the hypothesis that baseline platelet activation contributes to the magnitude of the inflammatory response to CPB. While causality cannot be inferred from the present design, the strength and consistency of this association align with the proposed upstream role of PAF in inflammatory amplification.

### 4.3. PAF and Postoperative Hemodynamic Instability

A key observation was the association between PAF and the postoperative vasoactive–inotropic requirements, particularly during the intermediate postoperative period. Importantly, this association was not observed immediately after surgery but became evident later, in a temporal pattern consistent with inflammation-driven vasoplegia, which typically develops hours after CPB, as cytokine release and endothelial dysfunction peak [14,15,16,17,30,31,32].

Inflammation-induced vasoplegia is characterized by endothelial dysfunction, cytokine release, and excessive nitric oxide production, leading to impaired vascular responsiveness [15,16,17]. PAF has been directly implicated in these processes through its effects on endothelial activation, leukocyte adhesion, and vascular smooth muscle signaling [12,13,16,28,29]. Prior studies have linked inflammatory activation to delayed vasoplegic syndromes following cardiac surgery [15,16,17]. Our findings extend this literature by suggesting that preoperative platelet-mediated inflammatory activation may predispose patients to exaggerated postoperative vascular dysfunction and increased hemodynamic support requirements.

### 4.4. Recovery Parameters and Length of Stay

The associations between preoperative PAF and global recovery parameters were more modest. Elevated PAF levels were associated with a trend toward prolonged mechanical ventilation and with a small but statistically significant increase in total hospital length of stay, whereas intensive care unit length of stay did not differ significantly between groups.

These outcomes represent integrated measures influenced by multiple clinical, organizational, and institutional factors. Previous studies have reported inconsistent associations between inflammatory biomarkers and recovery metrics such as ventilation duration or hospital length of stay, underscoring the multifactorial determinants of postoperative recovery [18,33,34,35,36]. The relatively limited magnitude of these associations in the present study suggests that while platelet-mediated inflammation contributes to early postoperative physiological instability, downstream recovery is shaped by a broader constellation of factors beyond inflammatory activation alone.

### 4.5. Acute Kidney Injury and Mortality

Although both PAF and CRP demonstrated univariate associations with adverse postoperative outcomes, neither biomarker remained independently associated with AKI or mortality after multivariable adjustment, as the OR for CRP (0.99, 95% CI 0.99–1.01) was not statistically significant (*p* = 0.32). Instead, age and body mass index emerged as the primary predictors of postoperative AKI.

These findings are consistent with prior literature indicating that AKI after cardiac surgery is largely driven by baseline patient susceptibility and renal reserve, with inflammatory activation acting as a secondary or amplifying factor rather than a direct causal mechanism [37,38,39,40,41,42]. Similarly, the modest discriminatory performance of PAF and CRP for mortality observed in our receiver operating characteristic analyses aligns with previous studies demonstrating that inflammatory biomarkers often lose prognostic power once established clinical risk factors are incorporated into multivariable models [43,44,45,46,47].

### 4.6. Clinical and Pathophysiological Implications

The present findings suggest that PAF and CRP should not be interpreted as stand-alone prognostic biomarkers but rather as indicators of a potential underlying biological vulnerability characterized by heightened inflammatory and vascular responsiveness to surgical stress. Our findings demonstrate that higher baseline PAF levels correlate with increased vasoactive–inotropic requirements during the critical intermediate postoperative period. This association follows a clear pathophysiological reasoning: PAF is known to promote endothelial dysfunction and vascular hyporesponsiveness—processes that are central to the development of CPB-associated vasoplegia.

Furthermore, we acknowledge that the field of major cardiac surgery is highly dependent on a multitude of key factors, including specific cardiac pathologies, baseline patient reserve, and operative variables such as bypass duration. However, from a pathophysiological standpoint, these results support a growing body of evidence implicating platelet-mediated inflammatory pathways in cardiovascular vulnerability and perioperative organ dysfunction [48,49,50,51,52]. By confirming that PAF’s role in the inflammatory cascade translates into increased clinical support requirements, this study provides the groundwork for personalized risk stratification and may help identify patients who would benefit from hypothesis-driven interventional studies. Ultimately, understanding these mechanistic pathways beyond conventional demographic or procedural variables, is a necessary step toward future therapeutic interventions designed to modulate the inflammatory burden of CPB.

### 4.7. Limitations

Several limitations to this study must be acknowledged. First, the retrospective, single-center design limits causal inference and generalizability. As an exploratory retrospective analysis, the sample size of 87 patients was insufficient to provide the statistical power required to identify independent correlations with outcomes such as acute kidney injury, ICU length of stay, or mortality, meaning that this study was statistically underpowered to detect anything but very large effect sizes for these rare outcomes. The small subgroup sizes limit the interpretability of categorical comparisons, which we addressed by utilizing multivariable regression analyses for the entire cohort. Furthermore, because no formal adjustments were made for multiple comparisons across these secondary clinical endpoints, the findings should be viewed as hypothesis-generating, and the risk of Type I error must be considered when interpreting the strength of these associations. The low number of events relative to the number of variables limits the statistical power and may lead to unstable estimates.

These clinical endpoints are influenced by a complex interplay of surgical, anesthetic, and institutional factors that may obscure the independent contribution of any single inflammatory mediator. Biomarkers and echocardiography parameters were measured at single time points, precluding the assessment of dynamic perioperative changes, and residual confounding cannot be excluded despite multivariable adjustment.

Despite these limitations, the strength of the association between preoperative PAF and the subsequent inflammatory and hemodynamic response provides a clear signal that this pathway warrants further, larger-scale prospective investigation to fully define its prognostic and therapeutic potential.

## 5. Conclusions

Preoperative platelet-activating factor is strongly associated with postoperative inflammatory burden and with increased postoperative hemodynamic support requirements following major cardiac surgery. While PAF and CRP are not independent predictors of adverse outcomes, they may help identify a biologically vulnerable phenotype characterized by exaggerated inflammatory and vascular responses to surgical stress. These findings are consistent with prior literature and support further investigation of platelet-mediated inflammatory pathways as potential targets for perioperative risk stratification and therapeutic intervention.

## Figures and Tables

**Table 1:** Baseline characteristics, perioperative variables, and preoperative platelet-activating factor (PAF) levels across postoperative C-reactive protein (CRP) strata in patients undergoing major cardiac surgery. NS = nonsignificant statistically. Bold/* = statistically significant.

Variable	CRP 0–10 mg/L (n = 3)	CRP 10–40 mg/L (n = 15)	CRP 40–200 mg/L (n = 59)	CRP > 200 mg/L (n = 6)	*p*-Value
**Patient Characteristics**					
Age (years)	70.3 ± 4.0	65.7 ± 4.6	64.6 ± 9.0	63.3 ± 12.8	NS
Weight (kg)	81.0 ± 11.5	85.3 ± 15.5	82.1 ± 17.6	91.7 ± 38.1	NS
Height (cm)	163.0 ± 11.3	168.8 ± 9.0	170.6 ± 8.7	152.0 ± 33.8	0.005 *
Body mass index (kg/m^2^)	30.4 ± 0.7	29.9 ± 4.6	28.1 ± 5.2	27.3 ± 6.5	NS
**Surgical Procedures**					
CABG, n%	0	3 (11.1%)	24 (88.9%)	1	NS
Valve surgery, n%	3 (6.9%)	9 (20.9%)	26 (60.5%)	5 (11.6%)	NS
Aortic surgery, n%	0	0	6 (100%)	0	NS
Combined procedures, n%	0	3 (30%)	7 (70%)	0	NS
**Perioperative Variables**					
Preoperative LVEF (%)	54.7 ± 5.0	52.3 ± 11.9	52.9 ± 10.6	43.7 ± 7.0	NS
Perfusion time (min)	124.3 ± 36.1	99.4 ± 62.7	90.4 ± 53.7	99.7 ± 29.3	NS
Aortic cross-clamp time (min)	98.3 ± 38.3	76.1 ± 52.7	73.0 ± 49.1	77.3 ± 26.5	NS
Mechanical ventilation (h)	5.7 ± 4.7	6.8 ± 4.7	6.2 ± 4.2	6.3 ± 6.9	NS
ICU stay (days)	2.7 ± 1.2	6.2 ± 10.5	3.3 ± 2.2	4.5 ± 1.8	NS
Hospital stay (days)	11.7 ± 3.5	13.0 ± 9.4	10.2 ± 3.3	9.8 ± 1.6	NS
**Biomarkers**					
Preoperative PAF (pg/mL)	**1428 ± 361**	**1830 ± 685**	**3369 ± 1012**	**4939 ± 2167**	**<0.001**

**Table 2:** Multivariable logistic regression analysis evaluating the association of platelet-activating factor (PAF), C-reactive protein (CRP), and clinical variables with postoperative AKI. β coefficients are expressed as log-odds. Odds ratios (ORs) are presented with 95% confidence intervals. Continuous variables were entered per unit increase as specified. Bold = statistically significant.

Variable	β (Log-Odds)	Odds Ratio (OR)	95% CI	*p*-Value
**PAF (per 1000 pg/mL)**	0.50	1.0005	0.999–1.001	0.20
**CRP (per 1 mg/L)**	−0.008	0.99	0.98–1.01	0.32
**Age (per year)**	0.107	**1.11**	1.02–1.21	**0.016**
**BMI (per kg/m^2^)**	0.126	**1.13**	1.01–1.28	**0.039**
CPB time (per min)	0.005	1.01	0.99–1.02	0.32

**Table 3:** Postoperative outcomes and complications stratified by peak postoperative C-reactive protein (CRP) levels.

Variable	Total (n = 87)	CRP 0–10 mg/L (n = 3)	CRP 10–40 mg/L (n = 15)	CRP 40–200 mg/L (n = 59)	CRP > 200 mg/L (n = 6)	*p*-Value
In-hospital Mortality, n (%)	6 (6.8%)	0 (0%)	1 (1.14%)	5 (5.74%)	0 (0%)	0.668
Acute kidney injury, n (%)	19 (21.8%)	1 (1.1%)	2 (2.3%)	15 (17.2%)	1 (1.1%)	0.334
Stroke, n (%)	1 (1.1%)	0 (0%)	1 (1.1%)	0 (0%)	0 (0%)	0.426
Arrhythmias, n (%)	33 (37.9%)	3 (3.8%)	8 (9.1%)	18 (20.6%)	4 (4.5%)	0.146
Anemia (%)	31 (35.6%)	2 (2.3%)	5 (5.7%)	21 (24.1%)	3 (3.4%)	0.307

## Data Availability

Data supporting the reported results can be found in Mega cloud through the following link: https://mega.nz/fm/YFERRRoC accessed on 11 February 2026.
